# Cardiovascular Outcomes in Hospitalized Patients with COVID-19: Does Age Really Matter?

**DOI:** 10.3390/jcdd12020041

**Published:** 2025-01-24

**Authors:** Alex Sotomayor-Julio, Manuela Escalante, Yorlany Rodas-Cortes, Andrea Alejandra Arteaga-Tobar, Andrea Valencia, Fernando Wyss, Roger Martín Correa, Paola Oliver, Wilbert Yabar Galindo, Jessica Mercedes, Alejandra Inés Christen, Iván Criollo, Juan Martin Brunialti, Carlos Eduardo Montenegro, Pedro Schwartzmann, Eglee Castillo, Freddy Pow Chong, Claudia Almonte, Cesar Herrera, Juan Esteban Gomez-Mesa

**Affiliations:** 1 Departamento de Cardiología, Fundación Valle del Lili, Cali 760032, Colombia; alex.sotomayor@fvl.org.co; 2 Faculty of Health Sciences, Universidad Icesi, Cali 760031, Colombia; manuela.escalante@fvl.org.co; 3 Centro de Investigaciones Clínicas, Fundación Valle del Lili, Cali 760032, Colombia; yorlanyrodas.fvl@gmail.com (Y.R.-C.); andrea.arteaga@fvl.org.co (A.A.A.-T.); andrea.valencia.or@fvl.org.co (A.V.); 4 Cardiology Department, Servicios y Tecnología Cardiovascular de Guatemala S.A.—CARDIOSOLUTIONS, Ciudad de Guatemala 01010, Guatemala; fernandowyss@gmail.com; 5 Cardiology Department, Hospital Nacional Alberto Sabogal Sologuren, Bellavista 07011, Peru; marcoflo2001@yahoo.es; 6 Cardiology Department, Hospital Nacional Arzobispo Loayza, Lima 15082, Peru; paolaoliver.re@gmail.com; 7 Cardiology Department, Hospital Nacional Guillermo Almenara Irigoyen, Lima 15033, Peru; yabar.galindo@gmail.com; 8 Cardiology Department, Hospital Nacional San Rafael, Santa Tecla 1501, El Salvador; draykmercedes@yahoo.com; 9 Cardiology Department, Hospital Presidente Perón, Buenos Aires B1872AWK, Argentina; christenalejandra@gmail.com; 10 Cardiology Department, Hospital Regional Arica, Arica y Parinacota 1000875, Chile; icll53@hotmail.com; 11 Cardiology Department, Hospital San Juan de Dios de la Plata, La Plata B1900, Argentina; jmbrunialti@gmail.com; 12 Cardiology Department, Pronto S. Cardiologico de PE. Prof. Luiz Tavares—PROCAPE, Recife 74970-240, Brazil; ce_montenegro@yahoo.com.br; 13 Cardiology Department, CLINICOR—Clínica Cardiológica LTDA, Goiânia 74075-040, Brazil; pedrovs.usp@gmail.com; 14 Cardiology Department, Centro Policlínico Valencia, Valencia 2001, Venezuela; egleecastillogonzalez@gmail.com; 15 Cardiology Department, Hospital Luis Vernaza, Guayaquil 090313, Ecuador; dr.freddypowchl@gmail.com; 16 Cardiology Department, Medicina Cardiovascular Asociada (MCA), Santo Domingo 10602, Dominican Republic; claudiaalmonte@hotmail.com; 17 Cardiology Department, Centro de Diagnóstico, Medicina Avanzada y Telemedicina (CEDIMAT), Santo Domingo 10216, Dominican Republic; cjherrera@cedimat.net

**Keywords:** age, COVID-19, cardiovascular disease, Latin America

## Abstract

Background: An advanced age elevates risk for COVID-19-related cardiovascular complications and mortality. This study analyzes cardiovascular comorbidities and outcomes in hospitalized COVID-19 patients across age groups to assess its impact. Methods: The CARDIO COVID 19-20 registry is a prospective, multicenter cohort study of hospitalized SARS-CoV-2 patients across 44 institutions in 14 Latin American countries. Patients were categorized into four age groups, Group 1: under 40 years, Group 2: 40 to 64 years, Group 3: 65 to 79 years, and Group 4: 80 years or older. Results: A total of 3260 patients were included. A total of 36.8% were women, and key comorbidities included overweight/obesity (49.7% [G1: 48.9%, G2: 56.3%, G3: 45.6%, G4: 32.7%]), and hypertension (49% [G1: 11.3%, G2: 40.3%, G3: 67.9%, G4: 80.4%]). Primary cardiovascular complications during hospitalization were arrhythmias (9.1% [G1: 3.4%, G2: 6.1%, G3: 14.9%, G4: 12.9%]), and acute heart failure (8.5% [G1: 3.6%, G2: 6.1%, G3: 12.1%, and G4: 15.2%]). In our cohort, 53.5% of the patients were admitted to the intensive care unit (G1: 49.2%, G2: 57%, G3: 55.3%, G4: 38.3%). In-hospital mortality rose significantly in patients aged 65 and older; G3: 334 (34.7%) and G4: 156 (45.6%) (*p* value: <0.001). Conclusions: In Latin American COVID-19 patients, older age correlates with more comorbidities, cardiovascular complications, and higher in-hospital and 30-day mortality, indicating age as an independent mortality factor.

## 1. Introduction

Severe acute respiratory syndrome coronavirus 2 (SARS-CoV-2) was identified and officially named coronavirus disease 2019 (COVID-19) by the World Health Organization (WHO) on 11 February 2020. Notably, this pneumonia caused by SARS-CoV-2, a virus distinct from all previously known coronaviruses, demonstrates significant infectivity but exhibits lower virulence when compared to severe acute respiratory syndrome (SARS) and the Middle East respiratory syndrome (MERS) [1].

The initial outbreak of COVID-19 infection posed a significant health emergency in Europe, particularly in Italy and Spain, as well as in the United States and Latin America. In response to this emergency, different countries implemented rigorous political, economic, and social measures to prevent or delay the spread of SARS-CoV-2 infection [2].

Data from the CDC COVID-NET database from the United States reveal that, throughout the peak of the pandemic, older patients had the highest hospitalization rates [3]. A literature review on predictors of severity in COVID-19 revealed that the hospitalization rate in older patients was the highest, and 62% of hospitalized patients in the United States in March 2020 were older than 55 years [4]. In addition, reports from the Public Health Agency of Canada stated that patients older than 45 have a higher prevalence of chronic diseases, such as diabetes and cardiovascular diseases [5].

Considering the high prevalence of cardiovascular disease in Latin American countries [6] and its direct relationship with mortality, the COVID-19 pandemic is expected to have a more severe negative impact in this population [4,7,8,9].

Based on these factors, the CARDIO COVID-19-20 registry, focused on hospitalized patients due to COVID-19, was established. The registry aimed to observe the baseline characteristics of patients, their cardiovascular comorbidities, cardiovascular complications, and clinical outcomes during hospitalization. This sub-analysis of the registry is focused on establishing the demographic characteristics, cardiovascular complications, and outcomes of patients hospitalized due to COVID-19 within four different age groups, with additional information during the 30-day follow-up period.

## 2. Methods

### 2.1. Study Oversight and Data Collection

**Figure 1:**
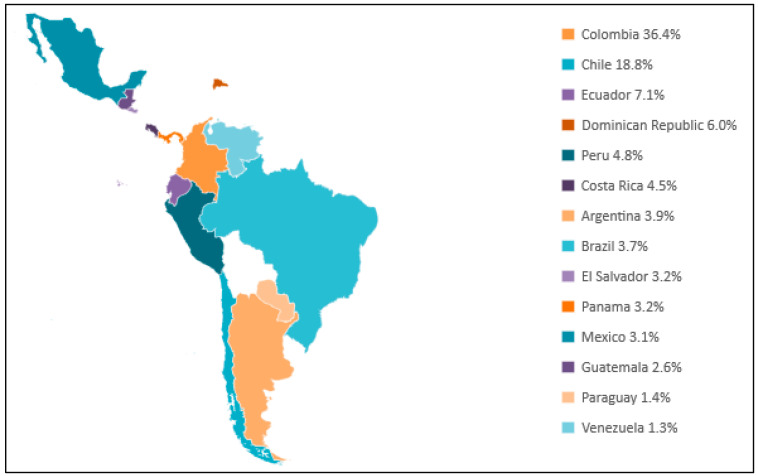
Distribution of patients by participating countries.

The full details of the study design, patient recruitment, eligibility criteria, and assessments were reported previously [10]. Briefly, the CARDIO COVID 19-20 is an observational, prospective, multicentric, and hospital-based registry that includes data from 44 hospitals in 14 Latin American countries (Argentina, Brazil, Chile, Colombia, Costa Rica, the Dominican Republic, Ecuador, El Salvador, Guatemala, Mexico, Panama, Paraguay, Peru, and Venezuela) [Figure 1]. The study protocol was approved by the Human Ethics Board Committee of the Fundación Valle del Lili (#1835) in Cali, Colombia. The registry was designed and coordinated by the Consejo Interamericano de Falla Cardíaca e Hipertensión Pulmonar (CIFACAH) of the Sociedad Interamericana de Cardiología (SIAC). Information (variables) was collected in the electronic database system RED Cap (Research Electronic Data Capture). This is a study based on data collected in routine clinical practice, and individual informed consent was not required; however, the anonymization of personal information was guaranteed for all sites.

Detailed information included demographics (age, sex, ethnicity, education, smoking status, and pregnancy status), comorbidities (cardiovascular and non-cardiovascular), previous cardiovascular treatment, signs and symptoms at admission, laboratory tests (admission and discharge), diagnostic tests and cardiovascular procedures performed during hospitalization, COVID-19 treatment during hospitalization, cardiovascular outcomes through hospitalization, in-hospital mortality and 30-day post-discharge mortality. The enrollment of patients was performed between 1 May 2020 and 30 June 2021.

### 2.2. Inclusion Criteria

(a) Patients older than 18 years with a confirmed diagnosis of COVID-19 according to guidelines provided by the WHO and institutional and/or local guidelines (World Health Organization. Diagnostic testing for SARS-CoV-2: interim guidance, 11 September 2020).
(b) Patients who required in-hospital management for more than 24 h related to COVID-19, or
(c) (Patients who died within the first 24 h after hospital admission due to COVID-19-related complications.

### 2.3. Outcomes

Clinical outcomes were assessed at the time of discharge and included in-hospital mortality and mortality within 30 days post-discharge. The primary outcome was “condition at discharge”, categorized as either alive or deceased, with a subset of the latter classified under “cardiovascular death”. The secondary outcomes were cardiovascular complications during hospitalization, intensive care unit (ICU) admission, and patient condition at the 30-day follow-up after being discharged from hospital. For the latter outcome, all patients were systematically followed to ascertain their vital status at the end of the 30-day post-discharge period and to identify re-hospitalization or mortality as a supplementary outcome measure. This follow-up was performed by phone, medical visit, or by reviewing clinical records.

### 2.4. Statistical Analysis

A descriptive analysis incorporating two methods to assess the normality of continuous variables: the Shapiro–Wilk test and the box-and-whisker diagram analysis. The data were presented as the median within the interquartile range (IQR). For categorical variables, the results were presented as absolute frequency and the corresponding percentage. The chi-square test was used to compare these variables. For quantitative variables, whose distributions did not conform to a normal form, nonparametric tests were applied for comparison. To examine the possible association between death during hospital stay and a broad spectrum of factors, such as demographic characteristics, history/comorbidities, vital signs, cardiovascular complications, treatments for COVID-19, clinical manifestations, and results of clinical and paraclinical tests, chi-square tests were used to assess the relationship between these variables. In addition, the inherent risk of multiple comparisons was addressed by implementing a false discovery rate (FDR) adjustment. This approach was adopted to safeguard against the possibility of obtaining significant results by mere chance due to repeated analysis of a diverse set of variables or groups. Poisson regression models with robust standard errors were used to calculate adjusted (aRR) and unadjusted (RR) rate ratios and 95% confidence intervals (CI). The goodness-of-fit of these models was assessed using Akaike’s information criterion (AIC) and applicable clinical considerations. For all tests and analyses, a statistical significance level of 0.05 was established. All statistical analyses were performed using R V.4.1.1 (R Foundation for Statistical Computing) through RStudio V.1.4.1717.

## 3. Results

For the age-group analysis in the present study, patients were grouped according to four age groups, as follows (Table 1):

- Group 1: Under 40 years (**G1**).
- Group 2: 40 to 64 years (**G2**).
- Group 3: 65 to 79 years (**G3**).
- Group 4: 80 years or older (**G4**).

### 3.1. Population Characteristics

A total of 3260 patients were included in the present study, of whom 36.8% were women; the median age of all patients in the registry was 61 years (interquartile range [IQR]: 48–71). A total of 32.2% of the patients included were referred from other institutions, and 67.8% were admitted directly from emergency or other hospital services. In the general population, the main comorbidities were overweight/obesity, 49.7%, hypertension, 49%, diabetes, 26.7%, and dyslipidemia, 13.8%. The most common cardiovascular treatments received before admission were angiotensin receptor blockers (24.7%), beta-blockers (13.3%), statins (12.2%), angiotensin-converting enzyme (ACE) inhibitors (11.0%), antiplatelets (10.8%), and diuretics (10.6%) (Table 1).

### 3.2. Clinical Findings on Admission

The most common clinical manifestations on admission were dyspnea (72.5%) followed by cough, fever, and fatigue. Younger patients (G1 and G2) presented others clinical manifestations such as fever, cough, myalgia, and constitutional symptoms, that is, symptoms of acute infection; however, concerning cardiovascular and respiratory clinical manifestations, they were similar in all the groups. Comparing the clinical manifestations profiles between older patients (G3 and G4), several similarities and differences emerge. Fever and cough are common and highly prevalent symptoms in both groups, though slightly higher in G3 (G3: 71.5% vs. G4: 49.4%). Dyspnea is notably common in both groups (G3: 67.4% and 71.6% in G4). However, G4 stands out, with a significantly higher occurrence of anorexia or appetite loss (24.6%) compared to G3 (16.3%). Myalgias and dysphagia appear to be more pronounced in the younger cohorts (G1, 34.8% and G2, 38.1%) than the older cohorts (G3, 30.2% and G4, 23.1%). The loss of taste and smell, neurological symptoms, and constitutional symptoms are more frequently reported in G1, 14.4% and G2, 7.0% than in G3, 4.7% and G4, 2.9%, indicating a variation in symptom presentation among age groups (Table 2).

### 3.3. Laboratory and Cardiovascular Tests

The troponin levels reported in this study correspond to those obtained at patient admission. Troponin was measured in 2086 patients (64%). Due to the nature of this multi-centric cohort, different troponin tests were performed, with ultrasensitive troponin I being the most used test across all groups (37.3%). The differences in troponin tests performed may have implications for the diagnostic accuracy of myocardial injury among patient groups. However, regardless of the location and type of troponin test, there was an increase in the mean troponin values within each group, which became progressively higher across age groups. For example, the ultrasensitive troponin I showed an increase in mean values as follows: G1: 0.0038 ng/mL, G2: 0.0077 ng/mL, G3: 0.0149 ng/mL, G4: 0.0285 ng/mL (*p*-value: 0.021). For all groups, the most common rhythm in the electrocardiogram was sinus rhythm. Older patients (G3 and G4) presented the highest prevalence of ventricular extrasystoles, whereas supraventricular extrasystoles were more prevalent in younger patients (G1). Atrial fibrillation (AF) was the second most predominant rhythm, showing an increase in its presentation with increasing age (Table 3).

In all groups, the main cardiovascular complications were cardiac arrhythmia, heart failure (HF), pulmonary embolism (PE), acute coronary syndrome (ACS), deep vein thrombosis (DVT), and myocarditis; all these complications showed variable incidences in the different age groups. Older patients (G3 and G4) had a higher propensity for these complications than younger patients (G1 and G2). Cardiac arrhythmia was the most common of all, accounting for 9.1% (296/3260) of patients, presenting with more prevalence in G3 and G4. The least common were myocarditis in 2.1% and DVT in 1.2% (40/3260) of patients. HF occurred in 8.5% (278/3620) (Table 4), with the most common hemodynamic presentation being the congestive profile.

### 3.4. Outcomes

Regarding outcomes in this cohort, 53.5% (1745/3620) Of the patients, 53.5% (1745/3260) were admitted to the intensive care unit (ICU), with the highest admission rate observed in G2 (40 to 64 years; 57%) and the lowest in G4 (80 years or older; 38.3%). Additionally, the length of stay in the ICU varied across groups, G4 patients had a median stay of 8 days (4.0, 15.0), compared to G2 (40–64 years) and G3 (56–79) with 10 days.

Among the 3260 hospitalized patients, 25.5% (831/3260) died during hospitalization, the highest prevalence of mortality was observed in G4 (45.6%) and the lowest in G1 (7.9%). In general, cardiovascular death represented 20.7% (172/831) of all deaths, and from these, patients in G2 (40 to 64 years) represented 21.8%, followed by G3 (65 to 79 years) with 19.5%. There were no statistically significant differences in the cause of death among the four groups. Concerning discharge conditions, a clear trend emerges where the proportion of patients who survived decreased with age, from 88.7% in G1 to 51.5% in G4 (Table 5).

### 3.5. Follow-Up 30 Days After Hospital Discharge

For the follow-up 30 days after discharge, we were able to contact 2046 patients and observed that 7.3% required rehospitalization and 2.6% of patients died, with 20.8% of these deaths attributed to cardiovascular causes. Mortality rates varied across age groups, being lower (1.0–1.3%) in younger groups (G1 and G2) and higher (4.9% and 6.2%) in older groups (G3 and G4). Cardiovascular factors contributed to 20.8% of those deaths. Conversely, non-cardiovascular causes dominated the mortality across all age groups. Rehospitalization rates within 30 days of discharge remained consistent across age groups (Table 6).

### 3.6. Univariate Analysis

Patients in G1 (younger patients) had a significant higher risk of dead during hospitalization when they were males, had overweight/obesity, coronary artery disease or prior HF, and were at lower risk if they had dyslipidemia or hypertension. Regarding cardiovascular complications, patients in G1 had a significantly higher mortality associated with acute HF, cardiac arrhythmia, and myocarditis compared to older patients (G4), with an unadjusted RR of 5.96 (95% CI: 2.90–12.22, *p* < 0.001), 9.21 (95% CI: 5.10–16.64, *p* < 0.001), and 6.66 (95% CI: 2.36–18.79, *p* < 0.001), respectively.

Patients in G2 had a significantly higher risk of in-hospital mortality as a primary outcome compared to other age groups, when they have a history of hypertension and HF with an unadjusted RR of 1.30 (95% CI: 1.07–1.59, *p* = 0.010) and 1.70 (95% CI: 1.15–2.52, *p* = 0.008), respectively. Regarding cardiovascular outcomes, mortality has a strong relationship with acute HF, cardiac arrhythmias, and pulmonary embolism and is less significant with myocarditis.

Patients in G3 had a significantly higher risk of in-hospital mortality as a primary outcome compared to G4, with an unadjusted RR of 1.88 (95% CI: 1.57–2.26, *p* < 0.001), 1.79 (95% CI: 1.50–2.14, *p* < 0.001) for acute HF, and cardiac arrhythmias.

G4 has the lowest OR values for primary and secondary outcomes, indicating weaker associations compared to younger age groups. For G3 and G4, the presence of comorbidities was not associated with a significantly increased risk of mortality (Table 7).

## 4. Discussion

### 4.1. Population Characteristics

With 3260 patients hospitalized for COVID-19 from 44 institutions of 14 countries, this is one of the largest cohort studies, with the greatest number of participating countries in Latin America. Most of the patients were men over 40 years, with overweight/obesity and hypertension as the major cardiovascular risk factors. These demographic patterns align with findings from Zhou et al. in Wuhan, China, where the median age was 56 and most patients were male (62%), with hypertension and diabetes being the most common comorbidities. Overweight/obesity was not reported [11]. In another retrospective cohort study in Korea by Song et al., which involved 5628 confirmed COVID-19 cases, a considerable proportion of patients were in the 50–59 age group, as in our study. Comorbidities were prevalent in almost half of the patients, including hypertension (21.3%) and diabetes (12.3%). These collective findings underscore the heightened risk of severe COVID-19 requiring hospitalization among male patients over 50 with an elevated body mass index and hypertension.

### 4.2. Cardiovascular Complications

Since the first clinical trials at the beginning of the COVID-19 pandemic, the ability of this disease to develop cardiovascular complications or to worsen underlying comorbidities was observed [5,12,13]. It was theorized that ACE-1 inhibitors and ARBs could be harmful in COVID-19, as increased ACE-2 activity could increase viral entry into cells [14,15,16]. Nevertheless, our patients continued treatment for their underlying pathologies unless there was a contraindication within hospitalization, such as cardiovascular deterioration or the requirement for vasopressor support. In our cohort, the use of ACE-1 inhibitors and ARBs did not show worsening cardiovascular outcomes (Table 1).

In our study, we could not observe a significant elevation in different troponins tests available in the participant centers, and this could not be related to worse results (Table 3), unlike other studies where the myocardial inflammatory process was associated with more symptoms and worse outcomes, as seen in the study by Guo T et al., where inflammation and elevated troponin levels were seen more in older patients (mean age 71.4 years), who had worse outcomes [8].

Likewise, severe SARS-CoV-2 disease was associated with coagulopathy, which represented an increased risk of thrombotic events such as DVP and PE, due to inflammation of the vascular, systemic, and pulmonary endothelium. Of the latter, PE represented the most frequent complication in different articles, and one of the most important risk factors for its development was age [5]. In our cohort, PE was the third and DVT was the fifth most common cardiovascular complications.

Confirmed cases of myocarditis were very low and predominantly in young patients (G1). Arterial thrombosis had the lowest incidence compared to other complications such as cardiac arrhythmias, decompensated HF, and ACS. In the multivariate analysis, we can appreciate that the presence of myocarditis in the young population (under 40) was highly associated with in-hospitalization mortality. In other registries and metanalysis, the relationship between mortality and myocarditis has been evidenced [13,14,15]; however, in the study by Annie FH et al. [13], the population was older than 50 years and had more comorbidities. In our univariate and multivariate analyses, the relationship of myocarditis was strongly associated with being younger than 40 years old.

In our study, patients hospitalized for COVID-19 had an increased prevalence of cardiovascular complications with increasing age, in which G4 was the most affected. These results are in line with those of Zhou et al., where multivariable regression showed increased odds of in-hospital death associated with older age (odds ratio 1.10, 95% CI 1.03–1.17 per year increase; *p* = 0.0043) [11], and in the Song et al. cohort, where univariate logistic regression analysis was performed, and the odds of mortality were higher in patients aged ≥ 60 years [17]. Likewise, the added risk for age groups > 45 years is relative to the age group of younger adults (18 to 44 years), and should not be interpreted as absolute risk [7,16].

### 4.3. Outcomes

Severe forms of disease increase with advancing age, as does the risk of mortality. Tajbakhsh A et al. reported that increasing age, especially in patients over 60 years of age, together with SARS-CoV-2 infection, is a risk factor for presenting more serious manifestations of the disease, especially in those with comorbidities [18]. In our cohort, we had similar findings. G4, with the highest prevalence of cardiovascular comorbidities, was the group with the highest cardiovascular complications, in-hospital mortality, and 30-day-follow-up mortality. However, it should be noted that this group did not represent the group with the highest admission to ICU. It can be seen in our cohort that for groups G1, G2, and G3, admission to the ICU corresponded to approximately 50%, while in the group over 80 years of age, it was only 38%.

This trend was not unique to the Latin American region; in the multicenter study by Guidet et al., 693 patients aged 80 years or older with acute respiratory failure with COVID-19 were compared with 1393 patients without COVID-19. COVID-19 patients were younger, less frail, less severe and had a lower SOFA score, but were treated more often with invasive mechanical ventilation (MV) and had a lower 30-day survival. In COVID-19 patients, the withholding and withdrawal of life-sustaining treatments was more frequent than in non-COVID-19 patients and 30-day survival was almost half that of non-COVID-19 patients [19].

Also, in the study by Giabicani et al., they found that, in 4671 patients with severe COVID-19, factors such as ICU burden, advanced age (≥ 80 years), frailty, and the severity of respiratory failure during the first 24 h, were associated with limitations of life-sustaining therapies [20].

This consideration has been reviewed from ethical and social points of view, where in times of scarcity, it may be justified to give priority to younger patients, in order to maximize the benefits for the greatest number of people, and because of the fair income that an elderly patient has already has [21].

After comparing the characteristics of COVID-19 patients of different ages and ICU admission, the longest length of stay may be related to the higher proportion of comorbidities in older patients. The increase in age associated with COVID-19 represents an important mortality risk factor; these patients are more likely to suffer from underlying comorbidities that can be exacerbated by the virus [4,5,7,8,16,22].

In general, the mortality rate was 25%, with the highest prevalence in G3 (34.7%), and in G4 (45.6%), with 20.7% of deaths being of cardiovascular causes. Similar findings were reported by studies by O’Brien et al., Song et al., Zhao et al., and Zhou et al. [5,11,17,22].

After discharge and at 30-day follow-up, the mortality was low (2.6%). This mortality risk was higher in the older (age ≥ 60 years) surviving patients than in the younger surviving patients. Cardiovascular-related deaths became more prevalent with increasing age, ranging from 0.0% in G1 to 20.0% in G4. Non-cardiovascular related deaths represented most cases, particularly in the older age. Rehospitalization rates within 30 days post-discharge also exhibited an age-related pattern, with the highest rate in G3 (10.4%) and a decrease in the younger group (4.3%). Statistical significance, as denoted by *p*-values, underscores the observed age-related variations in these outcomes.

This study can be compared with similar cohorts as the Wangs et al. study, where the survival probability of COVID-19 patients dramatically decreases when they present at older age [23].

Finally, in the population hospitalized for COVID-19 in LATAM, it was found that as age increases, a greater number of comorbidities, cardiovascular complications, and 30-day hospital mortality are observed. However, it is striking that, despite this increase in adverse outcomes, the older population (G3 and G4) required less admission and a lower length stay in the ICU, compared to younger groups (G1 and G2). This divergent relationship should be evaluated in detail at a later stage to analyze aspects that could lead to prioritizing admission to the ICU for populations with fewer comorbidities, with better prognosis, and with even younger patients.

### 4.4. Strengths and Limitations

Strengths: The CARDIO COVID 19-20 study presents a robust and comprehensive assessment of the impact of COVID-19 on hospitalized patients across Latin American countries. Its broad and varied dataset—which includes information from 44 hospitals across 14 countries—makes it stronger and improves the generalizability of the results. Because of the observational design ability to reduce bias, the effects of COVID-19 on cardiovascular health can be examined in a practical setting. During follow-up and data collection, strategies were adopted to reduce selection bias by defining cases in detail, identifying and recording comorbidities and complications during hospitalization, and defining 30-day follow-up times for patients discharged from hospital.

Robust data analysis and statistical evaluation strategies were used to perform univariate and multivariate analyses to avoid bias in the interpretation of the results.

Limitations: First, there may be an outcome bias in this cohort, as we lack follow-up and clinical outcome data for patients who were referred to other non-CARDIO COVID 19-20 registry institutions for continued inpatient treatment. The loss of data in the collection of patients can affect the results to some extent. Second, at that time, the potential of the virus to cause reinfection was not fully known, so this variable was not considered. Third, during the follow-up, the causes of mortality and rehospitalization were not specifically evaluated, leaving a gap on this point. Fourth, by the time the registry was conducted, vaccines were not available to be registered, and we could not evaluate their impact on outcomes. Finally, there will always be an inherent bias in registry information as not all patients are systematically included and there is always a risk of loss of information.

## 5. Conclusions

Related to COVID-19 in Latin America, as the population ages and accrues a higher burden of cardiovascular comorbidities during hospitalization, in-hospital mortality rates align with the premise that cardiovascular comorbidities become more prevalent with advancing age. Older age represents a risk factor, potentially leading to myocardial injury related to increased in-hospital stays and mortality after 30-day follow-up post-discharge when compared with younger patients.

## Figures and Tables

**Table 1:** Sociodemographic characteristics, comorbidities, and cardiovascular treatment on admission.

	Overall	Age				*p*-Value ^2^
		G1 (<40)	G2 (40–64)	G3 (65–79)	G4 (≥80)	
	**n = 3260 ^1^**	n = 417 ^1^	n = 1537 ^1^	n = 964 ^1^	n = 342 ^1^	
GENDER						<0.001
Female	1201 (36.8%)	187 (44.8%)	486 (31.6%)	367 (38.1%)	161 (47.1%)	
Male	2059 (63.2%)	230 (55.2%)	1051 (68.4%)	597 (61.9%)	181 (52.9%)	
COMORBIDITIES						
Overweight/obesity	1621 (49.7%)	204 (48.9%)	865 (56.3%)	440 (45.6%)	112 (32.7%)	<0.001
Hypertension	1596 (49.0%)	47 (11.3%)	619 (40.3%)	655 (67.9%)	275 (80.4%)	<0.001
Diabetes	869 (26.7%)	31 (7.4%)	407 (26.5%)	335 (34.8%)	96 (28.1%)	<0.001
Dyslipidemia	451 (13.8%)	8 (1.9%)	188 (12.2%)	201 (20.9%)	54 (15.8%)	<0.001
Smoking	438 (13.4%)	21 (5.0%)	187 (12.2%)	178 (18.5%)	52 (15.2%)	<0.001
Asthma/COPD	287 (8.8%)	30 (7.2%)	88 (5.7%)	109 (11.3%)	60 (17.5%)	<0.001
CKD	270 (8.3%)	14 (3.3%)	110 (7.1%)	98 (10.1%)	48 (13.8%)	<0.001
CAD	231 (7.1%)	4 (0.9%)	86 (5.6%)	104 (10.8%)	47 (14.2%)	<0.001
Heart failure	182 (5.6%)	10 (2.4%)	54 (3.5%)	75 (7.8%)	43 (12.6%)	<0.001
Atrial fibrillation	15 (3.5%)	2 (0.5%)	26 (1.7%)	57 (5.9%)	30 (8.8%)	<0.001
Previous stroke	102 (3.1%)	0 (0.0%)	23 (1.5%)	48 (5.0%)	31 (9.1%)	<0.001
CARDIOVASCULAR TREATMENT ON ADMISSION						
ARB	805 (24.7%)	23 (5.5%)	313 (20.4%)	331 (34.3%)	138 (40.4%)	<0.001
BB	432 (13.3%)	11 (2.6%)	142 (9.2%)	188 (19.5%)	91 (26.6%)	<0.001
Statin	398 (12.2%)	4 (1.0%)	141 (9.2%)	184 (19.1%)	69 (20.2%)	<0.001
ACE inhibitors	358 (11.0%)	12 (2.9%)	149 (9.7%)	141 (14.6%)	56 (16.4%)	<0.001
Antiplatelet	351 (10.8%)	10 (2.4%)	115 (7.5%)	160 (16.6%)	66 (19.3%)	<0.001
Diuretics	346 (10.6%)	14 (3.4%)	117 (7.6%)	147 (15.2%)	68 (19.9%)	<0.001
Anticoagulant	151 (4.6%)	6 (1.4%)	44 (2.9%)	64 (6.6%)	37 (10.8%)	<0.001
MRA	101 (3.1%)	9 (2.2%)	29 (1.9%)	39 (4.0%)	24 (7.0%)	<0.001
iSGLT-2	28 (0.9%)	1 (0.2%)	14 (0.9%)	10 (1.0%)	3 (0.9%)	0.2
ARNi	12 (0.4%)	1 (0.2%)	4 (0.3%)	7 (0.7%)	0 (0.0%)	0.5

ACE: angiotensin converter enzyme, ARB: angiotensin receptor blocker, ARNi: angiotensin receptor/neprilysin inhibitor, BB: beta blockers, CAD: coronary artery disease, CKD: chronic kidney disease, COPD: chronic obstructive pulmonary disease, iSGLT-2: sodium–glucose transport proteins inhibitor, MRA: mineralocorticoid receptor antagonist. ^1^ n (%); ^2^ Pearson’s chi-squared tests.

**Table 2:** Clinical manifestations and clinical findings.

Variable	Age					*p*-Value ^2^
	Overall, n = 3260 ^1^	G1 (18–39), n = 417 ^1^	G2 (40–64), n = 1537 ^1^	G3 (65–79), n = 964 ^1^	G4 (≥80), n = 342 ^1^	
CLINICAL MANIFESTATIONS						
Dyspnea	2365 (72.5%)	281 (67.4%)	1141 (74.2%)	698 (72.4%)	245 (71.6%)	0.048
Cough	2235 (68.6%)	279 (66.9%)	1124 (73.1%)	627 (65.0%)	205 (59.9%)	<0.001
Fever	2099 (64.4%)	298 (71.5%)	1067 (69.4%)	565 (58.6%)	169 (49.4%)	<0.001
Fatigue	1625 (49.8%)	196 (47.0%)	751 (48.9%)	495 (51.3%)	183 (53.5%)	0.2
Myalgias	1101 (33.8%)	145 (34.8%)	586 (38.1%)	291 (30.2%)	79 (23.1%)	<0.001
Anorexia/hyporexia	634 (19.4%)	68 (16.3%)	259 (16.9%)	223 (23.1%)	84 (24.6%)	<0.001
Neurological symptoms	504 (15.5%)	85 (20.4%)	247 (16.1%)	130 (13.5%)	42 (12.3%)	0.003
Constitutional symptoms	317 (9.7%)	58 (13.9%)	151 (9.8%)	80 (8.3%)	28 (8.2%)	0.009
Dysphagia	300 (9.2%)	41 (9.8%)	155 (10.1%)	87 (9.0%)	17 (5.0%)	0.029
Lost taste	213 (6.5%)	46 (11.0%)	111 (7.2%)	47 (4.9%)	9 (2.6%)	<0.001
Lost smell	223 (6.8%)	60 (14.4%)	108 (7.0%)	45 (4.7%)	10 (2.9%)	<0.001
CLINICAL FINDINGS						
Respiratory rate	22.0 (19.0, 28.0)	22.0 (18.0, 28.0)	22.0 (20.0, 28.0)	23.0 (19.0, 28.0)	22.0 (18.0, 28.0)	0.023
Heart rate	93.0 (80.0, 106.0)	100.0 (85.0, 112.0)	95.0 (82.8, 108.0)	90.0 (78.5, 104.0)	85.0 (75.0, 97.0)	<0.001
Systolic blood pressure	125.0 (112.0, 140.0)	120.0 (110.0, 130.0)	126.0 (114.0, 140.0)	126.0 (110.0, 143.0)	130.0 (118.0, 144.0)	<0.001
Diastolic blood pressure	75.0 (67.0, 83.0)	120.0 (110.0, 130.0)	126.0 (114.0, 140.0)	126.0 (110.0, 143.0)	130.0 (118.0, 144.0)	<0.001
Temperature	36.9 (36.2, 37.7)	37.0 (36.3, 37.9)	37.0 (36.3, 37.8)	36.8 (36.2, 37.5)	36.7 (36.2, 37.2)	<0.001
Oxygen saturation	91.0 (86.0, 95.0)	95.0 (90.0, 98.0)	91.0 (86.0, 95.0)	90.0 (84.0, 95.0)	90.0 (85.0, 94.0)	<0.001
Weight	75.0 (67.0, 86.0)	76.5 (68.0, 90.0)	80.0 (70.0, 90.0)	72.0 (65.0, 80.0)	68.0 (60.0, 75.0)	<0.001

^1^ n (%); ^2^ Pearson’s chi-squared tests.

**Table 3:** Complementary tests.

Variable	Age					*p*-Value ^2^
	Overall, n = 3260 ^1^	G1 (18–39), n = 417 ^1^	G2 (40–64), n = 1537 ^1^	G3 (65–79), n = 964 ^1^	G4 (≥80), n = 342 ^1^	
Troponin						
No troponin	1174 (36.0%)	182 (43.6%)	510 (33.2%)	348 (36.1%)	134 (39.2%)	
Troponin I	376 (11.5%)	51 (12.2%)	173 (11.3%)	112 (11.6%)	40 (11.7%)	
Troponin T	143 (4.4%)	14 (3.4%)	65 (4.2%)	49 (5.1%)	15 (4.4%)	
Ultrasensitive troponin I	1216 (37.3%)	132 (31.7%)	621 (40.4%)	345 (35.8%)	118 (34.5%)	
Ultrasensitive troponin T	351 (10.8%)	38 (9.1%)	168 (10.9%)	110 (11.4%)	35 (10.2%)	
Value						0.021
Troponin I ng/mL^2^	(n = 375) 0.0200 (0.0035, 0.1000)	0.0040 (0.0001, 0.0595)	0.0160 (0.0030, 0.1000)	0.0583 (0.0070, 0.1355)	0.0400 (0.0116, 0.1075)	
Troponin T ng/mL	(n = 143) 0.0206 (0.0080, 0.0500)	0.0400 (0.0051, 0.0468)	0.0188 (0.0060, 0.0500)	0.0197 (0.0101, 0.0450)	0.0314 (0.0150, 0.1459)	
Troponin I ultrasensitive ng/mL	(n = 1216) 0.0100 (0.0040, 0.0322)	0.0038 (0.0017, 0.0098)	0.0077 (0.0036, 0.0199)	0.0149 (0.0063, 0.0580)	0.0285 (0.0112, 0.1008)	
Troponin T ultrasensitive ng/mL	(n = 351) 0.0110 (0.0058, 0.0307)	0.0047 (0.0033, 0.0082)	0.0084 (0.0054, 0.0200)	0.0160 (0.0079, 0.0434)	0.0418 (0.0209, 0.1110)	
Electrocardiogram	1626 (49.9%)	167 (40.0%)	727 (47.3%)	538 (55.8%)	194 (56.7%)	<0.001
Sinus rhythm	1377 (88.5%)	149 (94.9%)	643 (93.1%)	436 (83.5%)	149 (80.1%)	
Ventricular extrasystoles	27 (1.7%)	0 (0.0%)	11 (1.6%)	12 (2.3%)	4 (2.2%)	
Supraventricular extrasystoles	9 (0.6%)	3 (1.9%)	4 (0.6%)	1 (0.2%)	1 (0.5%)	
Atrial fibrillation	84 (5.4%)	1 (0.6%)	15 (2.2%)	47 (9.0%)	21 (11.3%)	
Atrial flutter	7 (0.4%)	0 (0.0%)	1 (0.1%)	5 (1.0%)	1 (0.5%)	
Bundle block branch						<0.001
No	1370 (90.4%)	147 (95.5%)	643 (95.0%)	439 (87.1%)	141 (78.3%)	
Right	98 (6.5%)	5 (3.2%)	22 (3.2%)	47 (9.3%)	24 (13.3%)	
Left	47 (3.1%)	2 (1.3%)	12 (1.8%)	18 (3.6%)	15 (8.3%)	

^1^ n (%); ^2^ Pearson’s chi-squared tests. (%) Percentage troponin I ng/mL^2^. The analysis of the average troponin I value was conducted in 375 records due to one missing data point, while the total proportion of patients corresponds to 376 cases.

**Table 4:** Cardiovascular complications during hospitalization.

	Overall	Age				*p*-Value ^2^
		G1 (<40) ^1^	G2 (40–64) ^1^	G3 (65–79) ^1^	G4 (≥80) ^1^	
	n = 3260	n = 417	n = 1537	n = 964	n = 342	
Cardiac arrhythmia	296 (9.1%)	14 (3.4%)	94 (6.1%)	144 (14.9%)	44 (12.9%)	<0.001
Heart failure	278 (8.5%)	15 (3.6%)	94 (6.1%)	117 (12.1%)	52 (15.2%)	<0.001
Pulmonary embolism	126 (3.9%)	8 (1.9%)	61 (4.0%)	48 (5.0%)	9 (2.6%)	0.048
ACS	94 (2.9%)	0 (0.0%)	38 (2.5%)	42 (4.4%)	14 (4.1%)	<0.001
DVT	40 (1.2%)	0 (0.0%)	21 (1.4%)	11 (1.1%)	8 (2.3%)	0.030
Myocarditis	40 (1.2%)	4 (1.0%)	14 (0.9%)	13 (1.3%)	9 (2.6%)	0.066

ACS: acute coronary syndrome, DVT: deep vein thrombosis. ^1^ n (%); ^2^ Pearson’s chi-squared tests.

**Table 5:** Outcomes.

	Overall	Age				*p*-Value ^2^
		G1 (18–39) ^1^	G2 (40–64) ^1^	G3 (65–79) ^1^	G4 (≥80) ^1^	
	n = 3260	n = 417	n = 1537	n = 964	n = 342	
ICU admission	1745 (53.5%)	205 (49.2%)	876 (57.0%)	533 (55.3%)	131 (38.3%)	<0.001
Length of stay in ICU	10.0 (5.0, 18.0)	7.0 (3.0, 13.0)	10.0 (5.0, 18.0)	10.0 (5.0, 20.0)	8.0 (4.0, 15.0)	<0.001
DISCHARGE CONDITION n = 3260						<0.001
Alive	2304 (70.7%)	370 (88.7%)	1175 (76.5%)	583 (60.5%)	176 (51.5%)	
Dead	831 (25.5%)	33 (7.9%)	308 (20.1%)	334 (34.7%)	156 (45.6%)	
Referral	123 (3.8%)	14 (3.4%)	53 (3.5%)	46 (4.8%)	10 (2.9%)	
Missing		0	1	1	0	
CAUSE OF DEATH n = 831						0.2
Cardiovascular	172 (20.7%)	11 (33.3%)	67 (21.8%)	65 (19.5%)	29 (18.6%)	
Non-cardiovascular	659 (79.3%)	22 (66.7%)	241 (78.2%)	269 (80.5%)	127 (81.4%)	

^1^ n (%); ^2^ Pearson’s chi-squared tests. ICU: intensive care unit.

**Table 6:** Follow-up 30 days after hospital discharge.

	Overall	Age				*p*-Value ^2^
		G1 (18–39) ^1^	G2 (40–64) ^1^	G3 (65–79) ^1^	G4 (≥80) ^1^	
	n = 2046	n = 384	n = 1228	n = 629	n = 186	
CONDITION AT 30 DAYS POST-DISCHARGE						<0.001
Alive	1993 (97.4%)	309 (99.0%)	1031 (98.7%)	502 (95.1%)	151 (93.8%)	
Dead	53 (2.6%)	3 (1.0%)	14 (1.3%)	26 (4.9%)	10 (6.2%)	
CAUSE OF DEATH						0.4
Cardiovascular	11 (20.8%)	0 (0.0%)	5 (35.7%)	4 (15.4%)	2 (20.0%)	
Non-cardiovascular	42 (79.2%)	3 (100.0%)	9 (64.3%)	22 (84.6%)	8 (80.0%)	
REHOSPITALIZATION 30 DAYS AFTER DISCHARGE						0.4
NO	1831 (92.7%)	290 (95.7%)	934 (93.7%)	465 (89.6%)	142 (91.0%)	
YES	144 (7.3%)	13 (4.3%)	63 (6.3%)	54 (10.4%)	14 (9.0%)	
Missing	452	81	231	110	30	

^1^ n (%); ^2^ Pearson’s chi-squared tests.

**Table 7:** Univariate analysis.

	Age			
	G1 (<40)	G2 (40–64)	G3 (65–79)	G4 (≥80)
Male	3.02 (1.34–6.80, *p* = 0.008)	1.21 (0.97–1.52, *p* = 0.091)	1.36 (1.12–1.65, *p* = 0.002)	1.09 (0.87–1.38, *p* = 0.456)
COMORBIDITIES				
Dyslipidemia	0.00 (0.00–0.00, *p* < 0.001)	0.89 (0.64–1.23, *p* = 0.477)	1.01 (0.82–1.25, *p* = 0.916)	1.02 (0.74–1.39, *p* = 0.912)
Overweight/obesity	2.40 (1.17–4.92, *p* = 0.017)	1.14 (0.93–1.40, *p* = 0.212)	1.04 (0.88–1.24, *p* = 0.644)	0.81 (0.62–1.05, *p* = 0.113)
CAD	2.57 (0.43–15.34, *p* = 0.299)	0.98 (0.64–1.53, *p* = 0.946)	1.23 (0.96–1.58, *p* = 0.095)	1.03 (0.75–1.43, *p* = 0.839)
Hypertension	0.79 (0.25–2.48, *p* = 0.683)	1.30 (1.07–1.59, *p* = 0.010)	0.91 (0.76–1.09, *p* = 0.318)	0.84 (0.65–1.10, *p* = 0.204)
Heart Failure	2.63 (0.73–9.49, *p* = 0.141)	1.70 (1.15–2.52, *p* = 0.008)	1.30 (0.99–1.70, *p* = 0.060)	1.20 (0.89–1.63, *p* = 0.238)
CARDIOVASCULAR OUTCOMES				
Acute heart failure	5.96 (2.90–12.22, *p* < 0.001)	2.97 (2.39–3.70, *p* < 0.001)	1.88 (1.57–2.26, *p* < 0.001)	1.44 (1.12–1.85, *p* = 0.005)
Cardiac arrhythmia	9.21 (5.10–16.64, *p* < 0.001)	2.90 (2.32–3.63, *p* < 0.001)	1.79 (1.50–2.14, *p* < 0.001)	1.61 (1.27–2.05, *p* < 0.001)
Myocarditis	6.66 (2.36–18.79, *p* < 0.001)	1.07 (0.39–2.93, *p* = 0.896)	1.56 (0.94–2.61, *p* = 0.086)	0.97 (0.46–2.04, *p* = 0.944)
Pulmonary embolism	3.30 (0.95–11.48, *p* = 0.061)	2.04 (1.47–2.84, *p* < 0.001)	1.11 (0.76–1.61, *p* = 0.584)	1.48 (0.92–2.38, *p* = 0.107)
ACS	N/S	1.32 (0.77–2.27, *p* = 0.311)	1.32 (0.94–1.87, *p* = 0.112)	1.27 (0.79–2.02, *p* = 0.324)
DVT	N/S	1.43 (0.72–2.84, *p* = 0.302)	0.78 (0.30–2.07, *p* = 0.623)	1.10 (0.54–2.22, *p* = 0.793)
ICU admission	5.79 (2.28–14.71, *p* < 0.001)	5.69 (4.08–7.94, *p* < 0.001)	2.65 (2.13–3.30, *p* < 0.001)	1.88 (1.50–2.36, *p* < 0.001)

aRR (CI 95%, *p*-value): adjusted relative risk. Poisson regression models with robust standard errors. ACS: acute coronary syndrome, CAD: coronary artery disease, ICU: intensive care unit, N/S: no significance.

## Data Availability

The data that support the findings of this study are available from the corresponding author upon reasonable request.

## Supplementary Materials

The following supporting information can be downloaded at: https://www.mdpi.com/article/10.3390/jcdd12020041/s1, Table S1: Institutions That Recruited Patients. Membership of Cardio COVID 19–20 Team.

## Abbreviations

The following abbreviations are used in this manuscript:

SARS-CoV-2	Severe acute respiratory syndrome coronavirus 2
COVID-19	Coronavirus disease 2019
WHO	World Health Organization
SARS	Severe acute respiratory syndrome
MERS	Middle East respiratory syndrome
CDC	Centers for Disease Control and Prevention
CIFACAH	Consejo Interamericano de Falla Cardíaca e Hipertensión Pulmonar
SIAC	Sociedad Interamericana de Cardiología
REDCap	Research Electronic Data Capture
ICU	Intensive care unit
IQR	Interquartile range
FDR	False discovery rate
aRR	Adjusted rate ratio
RR	Rate ratio
CI	Confidence interval
AIC	Akaike’s Information Criterion
R	R (programming language for statistical computing)
G1	Group 1 (age group under 40 years)
G2	Group 2 (age group 40 to 64 years)
G3	Group 3 (age group 65 to 79 years)
G4	Group 4 (age group 80 years or older)
ACE	Angiotensin converter enzyme
AF	Atrial fibrillation
HF	Heart failure
PE	Pulmonary embolism
ACS	Acute coronary syndrome
DVT	Deep vein thrombosis
OR	Odds ratio
ARB	Angiotensin receptor blocker
LATAM	Latin America
