# Supplementary material for: Cardiovascular Outcomes in Hospitalized Patients with COVID-19: Does Age Really Matter?
Source: J Cardiovasc Dev Dis. 2025 Jan 24;12(2):41. doi: 10.3390/jcdd12020041 (PMC11856037; doi:10.3390/jcdd12020041)
Supplement: Supplementary file 1 [file jcdd-12-00041-s001.zip › jcdd-3363575-supplementary.pdf]

Table S1. Institutions That Recruited Patients.

Argentina	Hospital Presidente Perón
	Hospital San Juan de Dios de la Plata
	Hospital Universitario Fundación Favaloro
	Instituto de Cardiología J F Cabral
Brazil	CLINICOR—Clínica Cardiológica LTDA
	Hospital Ana Nery—HAN/SESAB
	Hospital Vera Cruz SA
	Pronto S. Cardiológico de PE. Prof. Luiz Tavares—PROCAPE
Chile	Hospital Dirección Previsional de Carabineros
	Clínica Dávila
	Hospital del Salvador
	Hospital Militar
	Hospital Regional Arica
	Instituto Nacional del Tórax
Colombia	Clínica Cardio VID
	Clínica de Occidente
	Clínica San Francisco
	Fundación Cardioinfantil
	Fundación Santa Fé
	Fundación Valle del Lili
	Hospital Universitario Erasmo Meoz
	Hospital Universitario San José Popayán
	San José de Buga
Costa Rica	Hospital San Vicente Paúl
	Hospital México
Dominican Republic	Centro de Diagnóstico, Medicina Avanzada y Telemedicina (CEDIMAT)
	Medicina Cardiovascular Asociada (MCA)
Ecuador	Hospital Luis Vernaza
	Centro de Atención Temporal "Quito Solidario"
	Hospital Eugenio Espejo
	Hospital Metropolitano
El Salvador	Hospital Nacional San Rafael
	Hospital El Salvador
Guatemala	Hospital Especializado de Villa Nueva
Mexico	Instituto Nacional de Cardiología—Ignacio Chávez
Panama	Hospital Santo Tomás
Paraguay	Instituto Cardiovascular Sanatorio Migone
	Instituto de Previsión Social
Peru	Hospital Nacional Guillermo Almenara Irigoyen
	Instituto Nacional Cardiovascular INCOR
	Hospital Nacional Alberto Sabogal Sologuren
	Hospital Nacional Arzobispo Loayza
Venezuela	Comité de Cardiología Tropical—Sociedad Venezolana de Cardiología
	Centro Policlínico Valencia

CARDIO COVID 19-20 TEAM:

PRINCIPAL INVESTIGATOR AND INSTITUTION

(ARG) Instituto de Cardiología J F Cabral	Maria Lorena Coronel
(ARG) Hospital Presidente Perón	Alejandra Ines Christen
(ARG) Hospital Universitario Fundación Favoloro	Paula Silva
(ARG) Hospital San Juan de Dios de la Plata	Juan Martin Brunialti
(BRA) CLINICOR - Clínica Cardiológica LTDA	Pedro Schwartzmann
(BRA) Hospital Ana Nery - HAN/SESAB	Luis Carlos Santana Passos
(BRA) Hospital Vera Cruz SA	Estevão Lanna Figueiredo
(BRA) Pronto S. Cardiológico de PE. Prof. Luiz Tavares - PROCAPE	Carlos Eduardo Montenegro
(CHI) Hospital Dirección Previsional de Carabineros	Franco Appiani Florit
(CHI) Clínica Dávila	Ricardo Enrique Larrea Gómez
(CHI) Hospital Militar	Fernando Verdugo Thomas
(CHI) Hospital Regional Arica	Iván Criollo
(CHI) Instituto Nacional del Tórax	Ricardo Ramírez Ramírez
(CHI) Hospital del Salvador	Víctor Rossel
(COL) Clínica del Occidente	Julián Lugo
(COL) Clínica San Francisco	Hugo Fernando Fernández
(COL) Fundación Cardioinfantil	Maria Juliana Rodríguez
(COL) Fundación Santa Fé	Andrés Buitrago
(COL) Fundación Valle del Lili	Noel Flórez
(COL) Hospital Universitario San José Popayán	Juan Isaac Ortíz
(COL) San José de Buga	William Millán Orozco
(COL) Clínica Cardio VID	Clara Inés Saldarriaga
(COL) Hospital Universitario Erasmo Meoz	Juan Carlos Ortega
(CRI) Hospital San Vicente Paúl	Daniel Quesada
(CRI) Hospital México	Andrés Ulate
(ECU) Centro de Atención Temporal "Quito Solidario"	Sylvia Sandoval
(ECU) Hospital Eugenio Espejo	Liliana Patricia Cárdenas Aldaz
(ECU) Hospital Metropolitano	Marlon Aguirre
(ECU) Hospital Luis Vernaza	Freddy Pow Chong
(ELS) Hospital Nacional San Rafael	Jessica Mercedes
(ELS) Hospital El Salvador	Jessica Mercedes
(GUA) Hospital Especializado de Villa Nueva	Armando Alvarado
(MEX) Instituto Nacional de Cardiología - Ignacio Chávez	Daniel Sierra
(PAN) Hospital Santo Tomás	Alexander Romero
(PAR) Instituto Cardiovascular Sanatorio Migone	Miguel Quintana
(PAR) Instituto de Previsión Social	Felipe Nery Gervacio Fernández
(PER) Hospital Nacional Alberto Sabogal Sologuren	Roger Martín Correa
(PER) Hospital Nacional Arzobispo Loayza	Paola Oliver
(PER) Instituto Nacional Cardiovascular INCOR	Francisco Chávez Sol Sol
(PER) Hospital Nacional Guillermo Almenara Irigoyen	Wilbert German Yabar Galindo
(RDO) Medicina Cardiovascular Asociada (MCA)	Claudia Almonte
(RDO) Centro de Diagnóstico, Medicina Avanzada y Telemedicina (CEDIMAT)	Cesar Herrera
(VEN) Comité de Cardiología Tropical - Sociedad Venezolana de Cardiología	Igor Morr
(VEN) Centro Policlínico Valencia	Eglee Castillo

ARG: Argentina; BRA: Brazil; CHI: Chile; COL: Colombia; CRI: Costa Rica; ECU: Ecuador: ELS: El Salvador; GUA: Guatemala; MEX: Mexico; PAN: Panama; PAR: Paraguay; PER: Peru; RDO: Dominican Republic; VEN: Venezuela.
